# White matter hyperintensities, cognitive decline and dementia risk in a community- dwelling cohort of northern Manhattan

**DOI:** 10.21203/rs.3.rs-9634268/v1

**Published:** 2026-06-04

**Authors:** Brenno Barreto, Huaizhi Ge, Mohammad Nafeli, Vanessa Guzman, Bonnie Levin, Stephanie Assuras, Janet De Rosa, Clinton Wright, Mitchell SV Elkind, Tatjana Rundek, Jose Gutierrez

**Affiliations:** Icahn School of Medicine at Mount Sinai; Columbia University; University of Miami Miller School of Medicine; Icahn School of Medicine at Mount Sinai; University of Miami Miller School of Medicine; Columbia University Irving Medical Center; Columbia University; National Institutes of Health; Columbia University; University of Miami Miller School of Medicine; University of Miami Miller School of Medicine

**Keywords:** White matter hyperintensities, Cognition, Dementia, Cerebral small vessel disease, Aging

## Abstract

**INTRODUCTION:**

White matter hyperintensities (WMH) are common markers of cerebral small vessel disease, but the cognitive impact of their regional distribution is not well understood.

**METHODS:**

We analyzed brain MRI and neuropsychological data from 1,290 stroke-free participants in the Northern Manhattan Study. WMH volumes were quantified by vascular territory, and associations with cognitive performance and dementia risk were evaluated.

**RESULTS:**

Higher anterior circulation WMH volume was associated with lower global cognition and poorer episodic memory, semantic memory, and processing speed at baseline and follow-up. Posterior circulation WMH predicted greater decline in executive function. Total WMH and anterior WMH volumes were associated with increased dementia risk.

**DISCUSSION:**

Total and regional WMH volumes can provide important information about cognitive outcomes and dementia risk, helping to understand which patients may have higher risk of worse cognitive outcomes. Then, tailored strategies can be used to individualize patient care and preserve overall brain health.

## 1. INTRODUCTION

White matter hyperintensities (WMH) are a common imaging biomarker for cerebral small vessel disease (CSVD)^1^ and are characterized by hyperintense signals in T2-weighted and fluid-attenuated inverse imaging recovery (FLAIR) magnetic resonance imaging (MRI). Among etiologies that can lead to WMH, ischemia has been shown to be a major cause of WMH because of hypoperfusion and blood-brain barrier disruption.^2,3^ WMH occurrence and progression are highly associated with older age and vascular risk factors, especially hypertension.^4,5^ WMH usually represents the very end of a continuum of white matter damage, with advanced imaging methods showing compromised white matter structural integrity very early in the disease process.^6^ The pathology correlates of WMH are highly heterogeneous, being usually characterized by demyelination and axonal loss.^7^

The increasing prevalence of WMH in the general population, due to population aging, has brought attention to the potential impact of WMH on the risk of vascular and, especially, cognitive outcomes.

Dementia has become a greater public health concern with an estimated projection of 152 million cases globally in 2050,^8^ and increasing efforts have been made to understand the role of WMH as a risk factor for cognitive decline and dementia. However, few studies managed to test such hypothesis in population-based, longitudinal cohorts, challenging the generalizability of their findings to multiethnic populations.^9–11^ Although these previous studies analyzed regional WMH burden associations with cognitive outcomes, results were conflicting. Additionally, there is no evidence in the current literature about whether WMH volumes by vascular territory or cerebral hemisphere reflect different patterns of association with cognitive performance and dementia risk in the general population.

In this study, we aimed to investigate the associations between WMH volumes and cognitive performance and dementia risk in an urban-based, multi-ethnic cohort of stroke-free individuals living in northern Manhattan.

## 2. METHODS

### 2.1 Patient sample

The Northern Manhattan Study (NOMAS) is a multi-ethnic, population-based cohort in an urban setting in northern Manhattan ongoing since 1993. Methods can be found elsewhere.^12^ Briefly, participants were randomly selected through random dial call and enrolled in the study as long as they had a telephone in home, permanent residency in one of the 5 zip codes in northern Manhattan, were 40 years old or more, and self-reportedly stroke-free. A total of 3298 participants were enrolled and followed up annually by telephone. Between 2003 and 2008, NOMAS participants were invited to undergo neuropsychological testing and brain MRI, constituting the NOMAS MRI substudy. Participants were eligible if they were 50 years or older, remained stroke-free (self-reported), and had no contraindications to undergo an MRI. A total of 1091 NOMAS participants were enrolled, and an additional 199 household members were invited to participate, for a total of 1290 participants in the MRI substudy. This study was conducted according to the Declaration of Helsinki. We followed the Strengthening the Reporting of Observational Studies in Epidemiology reporting guidelines.

### 2.2 Covariates adjudication

Demographic data was self-reported and obtained at the time of the MRI. Hypertension was defined as blood pressure ≥ 140/90 mmHg in two different measurements with a sphygmomanometer, self-report, or medication use at the time of the MRI. Diabetes mellitus was defined as fasting blood glucose ≥ 126 mg/dL, self-report, or medication use. Hypercholesterolemia was defined as total cholesterol ≥ 240 mg/dL, self-report, or medication use. Cigarette smoking was defined as current, past, or never at the time of enrollment. Apolipoprotein ε4 (ApoE4) carriers were identified through DNA samples from peripheral blood white cells using Hhal digestion and amplified by polymerase chain reaction.

### 2.3 Brain imaging acquisition

Baseline MRI was performed on a 1.5-T research MRI (Philips Healthcare, Best, the Netherlands) at Columbia University Irving Medical Center (CUIMC) following a standardized protocol. FLAIR images were acquired with a Multi-Slice Turbo Spin-Echo mode with a FOV of 250 mm; rectangular FOV of 80%; acquisition matrix of 192 × 133 scaled to 256 × 256 in reconstruction; slice thickness of 3 mm with no gap; flip angle of 90°; TR/TE of 5500 ms and 144 ms, respectively; and an inversion time of 1900 ms.

### 2.4 WMH volume quantification

WMH volume was obtained from FLAIR images through a semi-automated method with an in-house developed software as previously described.^13^ A Gaussian curve was fitted to map voxel intensity values after skull stripping of the images, and voxel intensity values above 3.5 standard deviation were labeled to be further quantified. WMH were visually inspected and manually corrected if needed. All labeled voxels were summed and multiplied to obtain the total WMH volume in cm^3^, and further log-transformed for normal distribution.

### 2.5 Other MRI variables acquisition

Total intracranial volume (TIV) was obtained through operator-guided manual removal of brain elements by tracing the dura mater within the cranium, maintaining the anterior and middle fossae, but excluding the posterior fossa and brainstem, as previously described.^14^

Covert brain infarcts (CBI) were evaluated using a combination of FLAIR image rim hyperintensity, shape, size and anatomic location to obtain a probabilistic diagnosis informed by pathology-derived MRI parameters by two vascular neurologists.^15,16^

### 2.6 Neuropsychological evaluation

Trained research staff performed extensive neuropsychological evaluation with study participants on the day of the MRI and during post MRI visits.

To calculate cognitive function, we clustered individual neuropsychological tests scores via factor analysis and used Scree plot of eigenvalues to determine the number of cognitive domains, and a factor > 0.3 was the criterion to include a certain test into a specific domain. We standardized individual neuropsychological test scores into Z-scores using cohort specific means and standard deviations at baseline, and we computed domain scores by averaging Z-scores from the individual tests related to a respective domain.

Neuropsychological tests clustering resulted in four major cognitive domains: executive function, episodic memory, semantic memory, and processing speed. Executive Function was obtained from the time to complete Color Trail Test Form 2 minus Form 1, and the sum of the Odd-Man Out test subtests 2 and 4 scores. Episodic Memory was derived from the subscores of a 12-word five-trial list-learning task (total immediate recall, delayed recall and delayed recognition). Semantic memory was assessed with the Boston Naming test (picture naming), the Animal Naming test (category fluency) and the C, F, L with English speakers and F, A, S for Spanish speakers (phonemic fluency). Processing Speed was obtained from the non-dominant hand Grooved Pegboard times, Color Trail Test Form 1 time, and Visual-Motor Integration test scores.^17^ Global cognition was obtained by averaging the Z-scores from all four domains.

### 2.7 Dementia adjudication

A team of neurologists and neuropsychologists with dementia expertise performed dementia adjudication using the criteria from the National Institute of Aging-Alzheimer’s Association (NIA-AA). At the time of MRI (2003–2008) and in addition to the neuropsychological tests, we obtained the clinical dementia-rating sum of boxes from the participant only. During the second visit (2008–2015), we added the Informant Questionnaire on Cognitive Decline in the Elderly and during the third visit (2016–2021), we added the Functional Activities Questionnaire. We obtained the Center for Epidemiologic Studies Depression Scale (CES-D) in all three visits. In addition to data obtained during the in-person neuropsychological evaluation, we reviewed the annual telephone follow up interview and electronic medical records available to us for mention of dementia, memory complaints or new use of medications to treat dementia. We also reviewed the Telephone Interview for Cognitive Status (TICS) obtained during the annual follow-up when available. For dementia adjudication, we prioritized data obtained from in-person neuropsychological and functional evaluations. For participants who did not come back for any post MRI visits or for those who did not come for the third visit, we relied on annual follow-up data, medical charts and TICS for cognitive adjudication. Dementia was diagnosed if there was evidence of: decreased performance in at least 1 cognitive domain; functional impairment; and lack of a psychiatric or other diagnosis that would explain the cognitive decline.^18,19^ Mild cognitive impairment was defined by three or more tests score (out of 13 tests) below 1.5 SD or a single cognitive domain < 1.5 SD but preserved functional status without alternative explanations.

### 2.8 Statistical analyses

Demographics were reported using descriptive statistics. Cross-sectional analysis investigating the association between baseline WMH volume and cognitive performance was performed using multivariate linear regression models.

We performed linear mixed models for repeated measures weighted by the inverse probability censoring weights (IPCW) to investigate the association between baseline WMH volume and longitudinal cognitive performance. Stabilized IPCW were accounted for possible nonrandom selection bias due to loss to follow-up over time.^20^ To estimate the IPCW, we fit pooled logistic models at each follow-up visit to predict the probability that a participant would remain at the study up until that visit. At each visit, we regressed a binary indicator for censorship based on baseline WMH, as well as time-invariant (sex, race/ethnicity, years of education, prevalent cardiovascular risk factors) and time-varying (age, cognitive performance during last visit) sociodemographic covariates. Stabilized weights were calculated as the marginal probability of each participant’s censorship status divided by each participant’s censorship status conditional to a set of time-invariant and time-varying covariates. Through this method, participants with a higher chance of being lost to follow-up would be weighted more heavily. This approach allows us to create a pseudopopulation where the censorship is independent of treatment.

To investigate the relation of WMH volume with dementia risk, we built risk models to obtain the hazard ratio and their 95% confidence interval using Cox proportional hazard regression models with robust sandwich error variance. Participants with dementia diagnosed at baseline neuropsychological evaluation (n = 16) were excluded from dementia risk analysis.

Lastly, we considered the modifying effect of ApoE4 status on total and regional WMH volumes. We fitted models with interaction terms between ApoE4 status and WMH volume to investigate their association with cognitive performance and dementia risk and further performed stratified analyses if a statistically significant interaction was found.

Our exposure variables consisted of total WMH volume and then by anatomical location, circulation, and hemispheres. Anatomical location was defined as subcortical, periventricular and posterior fossa (accounting for brainstem and cerebellum). Circulation was divided into anterior and posterior circulations. Hemispherical volumes were divided into right anterior, left anterior and posterior given the predominant blood supply in the posterior cortex by the vertebrobasilar system. Individuals with either right or left fetal posterior cerebral artery (PCA) had ipsilateral occipital WMH volume accounted as anterior circulation WMH.

Our outcomes were global cognition and its respective cognitive domains (executive function, episodic memory, semantic memory, and processing speed), and dementia risk.

All models were adjusted for sex, age, race/ethnicity, years of education, vascular risk factors, depression, ApoE4 carrier status, prevalent CBI at baseline, and TIV.

Two-sided p-values < 0.05 were considered statistically significant and < 0.10 for interactions. All statistical analyses were performed using R, version 4.5.0.

### 2.9 Standard Protocol Approvals, Registrations, and Patient Consents

The study was approved by the institutional review boards of Columbia University and the University of Miami. Written informed consent was obtained from all participants.

## 3. RESULTS

### 3.1 Sample characteristics

In our study, we included 1290 NOMAS participants (mean age 70.6 ± 8.9 years, 60.4% women, 65.7% Hispanic, mean follow-up time 8.85 years, Table 1). The mean WMH volume and standard deviation (SD) were 7.8 and 9.92 cm^3^, respectively. The crude incidence rate of dementia per 1,000 person-years and 95% CI was 20.02 (17.5–22.8). Our sample demographic characteristics were representative of the parent NOMAS cohort.^21^

### 3.2 WMH volume and cognitive performance at baseline

Total WMH volume was associated with lower global cognitive (adjusted β-estimate = −0.007 ± 0.002, p < 0.001), episodic memory (adjusted β-estimate = −0.010 ± 0.002, p < 0.001), semantic memory (adjusted β-estimate = −0.006 ± 0.002, p = 0.006), and processing speed (adjusted β-estimate = −0.011 ± 0.002, p < 0.001) scores (Table 2). When stratified by circulation, WMH volume in the anterior circulation was also associated with lower performance in global cognition (adjusted β-estimate = −0.009 ± 0.003, p < 0.001), episodic memory (adjusted β-estimate = −0.011 ± 0.004, p < 0.004), semantic memory (adjusted β-estimate = −0.010 ± 0.003, p < 0.003), and processing speed (adjusted β-estimate = −0.011 ± 0.004, p < 0.005). When further stratified by hemisphere, only right-sided anterior circulation WMH was associated with lower episodic memory (adjusted β-estimate = −0.062 ± 0.025, p = 0.01).

We assessed whether the interaction between WMH volume and ApoE4 status modified the association with global cognitive performance at baseline. ApoE4 was a significant modifier of the association of posterior fossa (interaction p = 0.05), and posterior circulation (interaction p = 0.04) WMH volumes with global cognitive score. In stratified analysis, posterior fossa WMH volume was associated with lower global cognitive score (adjusted β-estimate = −0.137 ± 0.069, p = 0.04) in ApoE4 carriers only.

### 3.3 WMH volume and longitudinal cognitive performance

Anterior and posterior circulation WMH volumes were independently associated with decreased global cognitive scores (Table 3). When analyzing by domain, anterior WMH was associated with poorer episodic and semantic memory, and processing speed while posterior circulation WMH volume was associated with poorer executive function. Posterior fossa WMH was associated with decreased global cognitive (adjusted β-estimate = −0.124 ± 0.056, p = 0.02) and executive function (adjusted β-estimate = −0.186 ± 0.072, p = 0.01) scores. ApoE4 status was not a significant modifier of the association between WMH volume and longitudinal global cognitive performance.

### 3.4 Baseline WMH volume and dementia risk

Greater baseline total WMH volume was associated with increased risk of dementia at follow-up (HR, 1.032; 95% CI, 1.019–1.045; Table 4). When analyzing by circulation, higher anterior circulation WMH volume was associated with increased risk of dementia (HR, 1.026; 95% CI, 1.006–1.047). No associations were found in analysis stratified by anatomical location or by hemisphere.

We assessed whether the interaction between WMH volume and ApoE4 modified the association with dementia risk. ApoE4 was a significant modifier of the interaction between subcortical (interaction p = 0.04), periventricular (interaction p = 0.01) and posterior fossa (interaction p = 0.08) WMH volumes and dementia risk. In stratified analysis, we found that among ApoE4 carriers, greater periventricular WMH volume was associated with increased risk of dementia (HR, 1.082; 95% CI, 1.007–1.163). No significant associations were found in stratified analysis between subcortical and posterior fossa WMH volumes and global cognitive score.

## 4. DISCUSSION

In this study, we investigated cross-sectional and longitudinal associations of WMH volume with cognitive performance and risk of dementia in an urban-based, multi-racial, stroke-free cohort of individuals living in northern Manhattan. We found that higher WMH volume was associated with lower performance across several cognitive domains, and with increased risk of dementia. The total WMH volume, as well as by anatomical site and circulation, plays an important role in cognitive decline and dementia risk.

Histopathological studies have shown that WMH can be highly heterogeneous, ranging from slight disentanglement of the matrix to varying degrees of myelin and axonal loss. Such heterogeneity may explain the clinicoradiological mismatch in cerebral small vessel disease.^22^ Furthermore, different biological pathways may lead to WMH occurrence within specific brain regions. Hypoxia has been investigated as a potential pathogenic mechanism in WMH, especially subcortical WMH, with capillary endothelial cells activation and expression of hypoxia-inducible factors.^23^ Blood-brain barrier (BBB) dysfunction also has been implied as a potential mechanism in WMH pathogenesis, being associated with both subcortical and periventricular WMH,^24^ possibly due to concentric collagen deposition in venular walls leading to venous stenosis with subsequent ischemic stress and BBB dysfunction.^25^ Hypertension can cause endothelial dysfunction, leading to impaired cerebral blood flow and BBB leakage. Given the cerebrovascular endothelial cells sensitivity to shear stress and hypoperfusion, their activation lead to a vicious circle, amplifying the white matter damage.^26^ Therefore, treatment and control of vascular risk factors, especially hypertension, is highly important to prevent the occurrence and progression of WMH and maintenance of brain health. Arterial health seems to be of special importance for cerebral anterior circulation, with hypertension being associated with higher baseline and longitudinal progression of WMH burden in all cerebral lobes except occipital.^11^ However, it is important to note that our findings were independent of hypertension, suggesting that the associations between WMH and cognitive decline and dementia risk are partially related to hypertension, but may also reflect other contributing mechanisms.

In our cross-sectional analysis, we found that total and anterior circulation WMH were associated with lower global cognitive function and performance across all domains except executive function. Our results partially align with previous evidence, where it was shown that higher WMH load at the parietal lobe was associated with worse executive function, verbal and non-verbal memory,^27^ and WMH in parietal and temporal lobes was associated with worse memory performance.^28^ These findings could be a result of the WMH affecting strategic subcortical structures and tracts. For example, episodic memory was previously found to be associated with the uncinate, and inferior and superior longitudinal fasciculi integrity, as well as the dorsal cingulum bundle.^29^ These tracts provide connections between the temporal, parietal and frontal lobes.^30–33^ Semantic memory was previously associated with temporal and inferior parietal lobes.^34^ Processing speed was associated with integrity of the white matter connecting the parietal and temporal cortices, and superior longitudinal fasciculus activity as seen in voxel-based morphometry.^35^ Since these tracts and structures are all supplied by the anterior circulation, impairment to their integrity could partially explain our results. When analyzing by hemisphere, the right anterior circulation WMH volume was associated with lower episodic memory. This finding could be explained based on previous evidence showing a more prominent connectivity network within the right hemisphere during successful episodic memory retrieval.^36^

In our longitudinal analysis, we found that total and anterior circulation WMH volume remained associated with decreased global cognitive function and decreased performance in episodic memory, semantic memory, and processing speed. Additionally, we found that posterior fossa and posterior circulation WMH volumes were associated with decreased global cognition and executive function during follow-up. These findings may be interpreted as somehow surprising given the historical association of frontal lobe lesions and executive function,^37^ and previous evidence linking higher WMH burden in the frontal and parietal lobes to executive dysfunction.^27^ However, it has been shown that WMH affecting the anterior thalamic radiation, which connects the anterior and midline nuclear groups of the thalamus with the frontal lobe, caused a negative impact in executive function.^38^ The anterior thalamus receives its blood supply from tuberothalamic branches of the posterior communicating artery (Pcomm), with tuberothalamic infarcts being also associated with executive dysfunction.^39^ Therefore, posterior circulation WMH especially affecting the anterior thalamus could be the biological explanation behind the executive dysfunction found in this association. Furthermore, smaller midbrain and locus coeruleus volumes were previously associated with worse executive function in individuals with MCI,^40^ and white matter disruption affecting these structures could be the reason why posterior fossa WMH volume was also associated with worse executive function. Posterior WMH accumulation has been previously associated with AD-related degenerative mechanisms.^41^ Our findings that higher posterior circulation and posterior fossa WMH volumes are associated with worse global cognitive function could be a result of lower executive function, as global cognition is calculated as an average of all four domains. It may also represent a prodromal finding in individuals who have not yet converted to AD but are already experiencing global cognitive decline, with a possible additional effect being conferred to ApoE4 carriers, in whom posterior fossa WMH volume was associated with lower global cognitive score at baseline in our stratified analysis. Lastly, a potential pathological mechanism for driving such associations could be outward arterial remodeling. Outward remodeling of intracranial arteries may decrease the cerebral vasculature impedance, leading to a higher transmission of pulsatile energy to the downstream circulation and potentially damaging brain parenchyma. As previously described, outward remodeling of the first segments of medial and anterior cerebral arteries were associated with higher WMH volumes.^42^ It is possible that the same applies to the posterior cerebral circulation, especially considering previous findings showing higher prevalence of histopathological markers of outward remodeling in the posterior circulation arteries when compared to anterior circulation arteries.^43^ Additionally, previous evidence has shown that larger left PCA/PComm mean diameters were associated with worse global cognitive decline at follow-up when compared to individuals with average diameters.^17^

Total and anterior circulation WMH volumes were associated with increased risk of dementia at follow-up. Our results align with previous evidence, where higher baseline and progression of parietal lobe WMH volume were independent predictors of progression to Alzheimer’s disease in a cohort of non-demented individuals.^10^ Similarly, previous research has shown that total WMH burden and progression was associated with conversion to both AD and dementia in individuals with mild cognitive impairment regardless of their amyloid status.^11^ It is important to note that we did not find any association between subcortical or periventricular WMH volumes with cognitive decline or dementia risk. These results should highlight the importance of looking into patterns of WMH distribution more based on vascular territories and its possible impact on cognition and dementia risk rather than between subcortical versus periventricular as a general approach, despite the known different pathological pathways for occurrence and progression of WMH between both regions. However, when accounting for ApoE4 status, the anatomical location may play a role in dementia risk. In our stratified analysis, among ApoE4 carriers, periventricular WMH volume was associated with an increased risk of dementia. ApoE4 is an emerging risk factor for CSVD and has been associated with increased risk of vascular dementia previously in literature.^44,45^

Our study has important strengths. The population-based, multi-ethnic, stroke-free cohort in an urban setting allows us to generalize our results to the general population. Our unique analysis broadens our perspective by not limiting the subdivision of brain regions between subcortical versus periventricular WMH but also considering vascular territories. Investigating WMH distribution among vascular territories highlights the importance of controlling vascular risk factors, especially hypertension, to prevent WMH progression in brain lobes primarily supplied by the anterior circulation (i.e., frontal, parietal, and temporal), which is distinctly affected by hypertension.

Our study also presents limitations. The lack of repeat imaging prevents us from considering the WMH progression over time and how this change may affect cognition and dementia risk. Furthermore, we understand that the division between anterior and posterior circulation may be somewhat arbitrary, especially when delimiting the posterior parietal and temporal lobes, where blood supply is more equally provided by both medial and posterior cerebral arteries.

In summary, our study provides novel evidence that WMH burden, when analyzed under a vascular territory division, is associated with cross-sectional and longitudinal cognitive decline among several cognitive domains and increased dementia risk in an urban-based, multi-ethnic cohort of stroke-free individuals in northern Manhattan. Total WMH volume, and more specifically, WMH volume within the brain region supplied by the anterior circulation, showed the most consistent associations and were the only exposures that accounted for the increased risk of dementia. These findings may help identify which patients can present an increased risk of dementia and individualize patient care through tailored strategies to prevent worse cognitive outcomes and preserve overall brain health.

## Supplementary Material

Supplementary Files

This is a list of supplementary files associated with this preprint. Click to download.

- WMHCogBrainImagingTables.docx

## Figures and Tables

**Table 1:** Demographics

Age (in years, mean ± SD)	NOMAS (N = 1290)
	70.6 ± 8.9
Female sex (%)	780 (60.4)
Race/ethnicity	
Non-Hispanic White	191 (14.8)
Non-Hispanic Black	223 (17.3)
Hispanic	847 (65.7)
Other	29 (2.2)
Education (in years, mean, median, IQR)	9.7, 10, 5–14
Hypertension at baseline MRI (%)	1010 (78.3)
Diabetes at baseline MRI (%)	329 (25.5)
Hypercholesterolemia at baseline MRI (%)	1047 (81.2)
Current smoking at baseline MRI (%)	151 (11.7)
ApoE 4 carrier (%)	312 (24.2)
White matter hyperintensity volume (mean ± SD, median, IQR)	7.8 ± 9.92, 4.2, 2.4–8.6
Follow-up time (in years, mean, median, IQR)	8.85, 9.64, 5.06–12.6
Covert brain infarct at baseline MRI (%)	237 (18.3)
Crude incidence rate (per 1,000 person-years) (95% CI)	
Dementia	20.02 (17.5–22.8)

Abbreviations: IQR, interquartile range; CI, confidence interval.

**Table 2:** Prevalent log-transformed white matter hyperintensities (WMH) with cognitive performance at baseline (cross-sectional)

		Global	Executive Function	Episodic Memory	Semantic Memory	Processing Speed
		β-estimate (SE)				
Total WMH		−0.007 (0.002)	−0.004 (0.003)	−0.010 (0.002)	−0.006 (0.002)	−0.011 (0.002)
		p < 0.001	p = 0.15	p < 0.001	p = 0.006	p < 0.001
M1	Subcortical WMH	−0.013 (0.009)	−0.002 (0.013)	−0.015 (0.014)	−0.012 (0.012)	−0.015 (0.014)
		p = 0.14	p = 0.88	p = 0.28	p = 0.31	p = 0.28
	Periventricular WMH	−0.007 (0.004)	−0.002 (0.007)	−0.010 (0.007)	−0.009 (0.006)	−0.009 (0.007)
		p = 0.14	p = 0.74	p = 0.14	p = 0.13	p = 0.15
	Posterior Fossa	−0.049 (0.037)	−0.096 (0.054)	−0.009 (0.055)	−0.066 (0.049)	−0.026 (0.055)
		p = 0.17	p = 0.07	p = 0.86	p = 0.17	p = 0.63
M2	Anterior circulation	−0.009 (0.003)	−0.003 (0.004)	−0.011 (0.004)	−0.010 (0.003)	−0.011 (0.004)
		p = 0.001	p = 0.48	p = 0.004	p = 0.003	p = 0.005
	Posterior circulation	−0.043 (0.038)	−0.111 (0.057)	0.002 (0.058)	−0.063 (0.051)	−0.001 (0.058)
		p = 0.26	p = 0.05	p = 0.96	p = 0.21	p = 0.98
M3	Right anterior circulation	−0.033 (0.017)	−0.035 (0.024)	−0.062 (0.025)	−0.0002 (0.022)	−0.027 (0.025)
		p = 0.05	p = 0.14	p = 0.01	p = 0.99	p = 0.28
	Left anterior circulation	0.011 (0.018)	0.029 (0.025)	0.035 (0.026)	−0.019 (0.023)	−0.011 (0.026)
		p = 0.54	p = 0.25	p = 0.17	p = 0.39	p = 0.65
	Posterior circulation	−0.025 (0.038)	−0.094 (0.056)	0.038 (0.057)	−0.062 (0.051)	0.018 (0.058)
		p = 0.51	p = 0.09	p = 0.50	p = 0.22	p = 0.75

Models adjusted for age, sex, race/ethnicity, years of education, prevalent hypertension, diabetes, smoking, hypercholesterolemia, depression, Apolipoprotein ε4 (ApoE4) carrier status, covert brain infarcts, and total cranial volume.

P < 0.05, P = 0.05–0.1.

**Table 3:** Prevalent log-transformed white matter hyperintensities (WMH) and cognitive decline (longitudinal)

		Global	Executive Function	Episodic Memory	Semantic Memory	Processing Speed
		**β-estimate (SE)**				
Total WMH		−0.009 (0.002)	−0.003 (0.004)	−0.012 (0.003)	−0.010 (0.004)	−0.014 (0.004)
		p < 0.001	p = 0.39	p < 0.001	p = 0.008	p < 0.001
M1	Subcortical WMH	−0.021 (0.014)	−0.010 (0.019)	−0.033 (0.019)	−0.025 (0.021)	−0.013 (0.020)
		p = 0.13	p = 0.58	p = 0.07	p = 0.22	p = 0.52
	Periventricular WMH	−0.005 (0.006)	0.008 (0.009)	−0.005 (0.009)	−0.009 (0.010)	−0.016 (0.009)
		p = 0.41	p = 0.36	p = 0.58	p = 0.35	p = 0.08
	Posterior Fossa	−0.124 (0.056)	−0.186 (0.072)	−0.095 (0.073)	−0.136 (0.081)	−0.064 (0.076)
		p = 0.02	p = 0.01	p = 0.19	p = 0.09	p = 0.39
M2	Anterior circulation	−0.011 (0.004)	0.002 (0.005)	−0.014 (0.005)	−0.015 (0.006)	−0.016 (0.006)
		p = 0.006	p = 0.76	p = 0.009	p = 0.01	p = 0.005
	Posterior circulation	−0.151 (0.059)	−0.234 (0.076)	−0.103 (0.077)	−0.155 (0.085)	−0.089 (0.080)
		p = 0.009	p = 0.002	p = 0.18	p = 0.06	p = 0.26
M3	Right anterior circulation	−0.030 (0.027)	−0.024 (0.036)	−0.050 (0.036)	0.003 (0.040)	−0.033 (0.038)
		p = 0.28	p = 0.50	p = 0.16	p = 0.94	p = 0.39
	Left anterior circulation	−0.004 (0.028)	0.027 (0.037)	0.011 (0.037)	−0.032 (0.041)	−0.023 (0.039)
		p = 0.88	p = 0.46	p = 0.77	p = 0.43	p = 0.55
	Posterior circulation	−0.137 (0.059)	−0.232 (0.076)	−0.083 (0.077)	−0.150 (0.085)	−0.077 (0.081)
		p = 0.01	p = 0.002	p = 0.27	p = 0.07	p = 0.34

Models adjusted for age, sex, race/ethnicity, years of education, prevalent hypertension, diabetes, smoking, hypercholesterolemia, depression, Apolipoprotein ε4 (ApoE4) carrier status, covert brain infarcts, and total cranial volume.

P < 0.05, P = 0.05–0.1.

**Table 4:** Prevalent log-transformed white matter hyperintensities (WMH) and dementia risk

		HR, 95% confidence interval
**M0**	**Total WMH**	**1.032 (1.019, 1.045)**
M1	Subcortical WMH	1.034 (0.957, 1.117)
	Periventricular WMH	1.024 (0.987, 1.062)
	Posterior Fossa	0.951 (0.598, 1.514)
M2	Anterior circulation	1.026 (1.006, 1.047)
	Posterior circulation	1.008 (0.619, 1.641)
M3	Right anterior circulation	1.089 (0.912, 1.299)
	Left anterior circulation	1.015 (0.850, 1.212)
	Posterior circulation	0.887 (0.542, 1.451)

Models adjusted for age, sex, race/ethnicity, years of education, prevalent hypertension, diabetes, smoking, hypercholesterolemia, depression, Apolipoprotein ε4 (ApoE4) carrier status, covert brain infarcts, and total cranial volume.

P < 0.05, P = 0.05–0.1.

## Data Availability

Data is available upon request to corresponding author.
